# The Surgical Repair of an Avulsion Dog Bite and Scar Revision in Highly Melanated Skin: A Case Report

**DOI:** 10.7759/cureus.74367

**Published:** 2024-11-24

**Authors:** Reena Sheth, Mrudangi Thakur

**Affiliations:** 1 Foundational Sciences, Nova Southeastern University Dr. Kiran C. Patel College of Osteopathic Medicine, Fort Lauderdale, USA; 2 Plastic and Reconstructive Surgery, Middletown Aesthetics and Plastic Surgery, Middletown, USA

**Keywords:** dark skin, dog bite, facial cosmetic surgery, full thickness skin graft, highly melanated, high melanin content, plastic and reconstructive surgery, scar appearance, scar site, surgical case report

## Abstract

Dog bites are one of the most common injuries in the United States, with varying presentations such as avulsion injuries and lacerations, and they range from a single bite to multiple bites in a victim. The severity of the dog bite is often the biggest factor in determining the treatment course. This report discusses the treatment of a 60-year-old male with severe facial avulsion trauma from a dog bite. The initial treatment involved debridement and grafting using salvaged tissue. Due to the high risk of cosmetic complications such as hypertrophic scarring and keloids in individuals with high melanin content, a secondary scar revision was planned to enhance aesthetic outcomes. The use of salvaged tissue for grafting, followed by scar revision, proved effective in minimizing disfigurement and improving patient satisfaction. This approach was chosen due to the heightened risk of keloid and hypertrophic scarring in patients with high melanin content, highlighting the importance of tailored surgical strategies. The findings suggest that using salvaged tissue combined with strategic scar revision can significantly improve cosmetic and functional results in complex dog bite injuries.

## Introduction

It is estimated that 45 million dog bites happen in the United States yearly in both adults and children [1]. Additionally, there has been a 47% increase in dog bites after the coronavirus disease 2019 (COVID-19) pandemic, with an increasing severity indicating the need for complex surgical management such as skin grafting or nerve repair with longer hospital stays for patients. The prevalence of dog bites post-COVID-19 with increasing severity is linked to a functional, cosmetic, and psychological impact on these patients, which must be further explored through research [2]. Oftentimes, due to the nature of the contamination of the wound, amoxicillin-clavulanate is the first-line treatment for dog bites. In the majority of children and adults injured by dogs, there has been an emphasis on function over aesthetics, which has negatively impacted patients' lives.

The lip is the most common site of injury from a dog bite to the face [3]. Avulsive dog bites are the most severe type in terms of dog bites to any part of the human body, with associated loss and tearing of the skin and underlying structures [4]. Common treatments for dog bites include irrigation, antibiotic prophylaxis with amoxicillin-clavulanate, and healing by secondary intention, or primary intention if cosmetically favorable, on the patient's face or a gaping wound [5]. Based on the type of the animal bite, rabies post-exposure prophylaxis and euthanasia of the offending animal can be considered; however, rabies protocol for animal bites vary based on a variety of factors, such as the species, availability of the animal for quarantine, the home environment of the animal, previous vaccinations, and whether the bite was provoked or unprovoked [6,7]. In the case of a domesticated pet with documentation of the rabies vaccine, the animal is observed for 10 days to see if signs of rabies are present. If after 10 days the animal remains healthy, there is no requirement for post-exposure prophylaxis of rabies for the patient [8].

There is limited research on dog bites in individuals with high melanin content and the types of cosmetic surgical options available for facial reconstruction of patients with this complexion. Traditional medical training in the United States has a limited but growing focus on the treatment of patients with pigmented skin and how scar revision and cosmetic outcomes work in these patients. Keloids and hypertrophic scarring occur in about 4.5-16% of the population, with a high prevalence in patients with high melanin content in their skin [9]. Thus, these aspects should be borne in mind when attempting reconstructive surgery on highly melanated individuals for a better cosmetic outcome. Although there are multiple treatment modalities available for keloids and hypertrophic scars, they still prove difficult to eradicate [9]. Furthermore, there is a negative correlation between quality of life and the presence of keloids and hypertrophic scars in patients, which highlights the significance of considering these complications when dealing with cosmetic procedures in individuals with high melanin content, especially those involving the face [10].

Written consent for publication of this study was obtained from the patient.

## Case presentation

**Figure 1:**
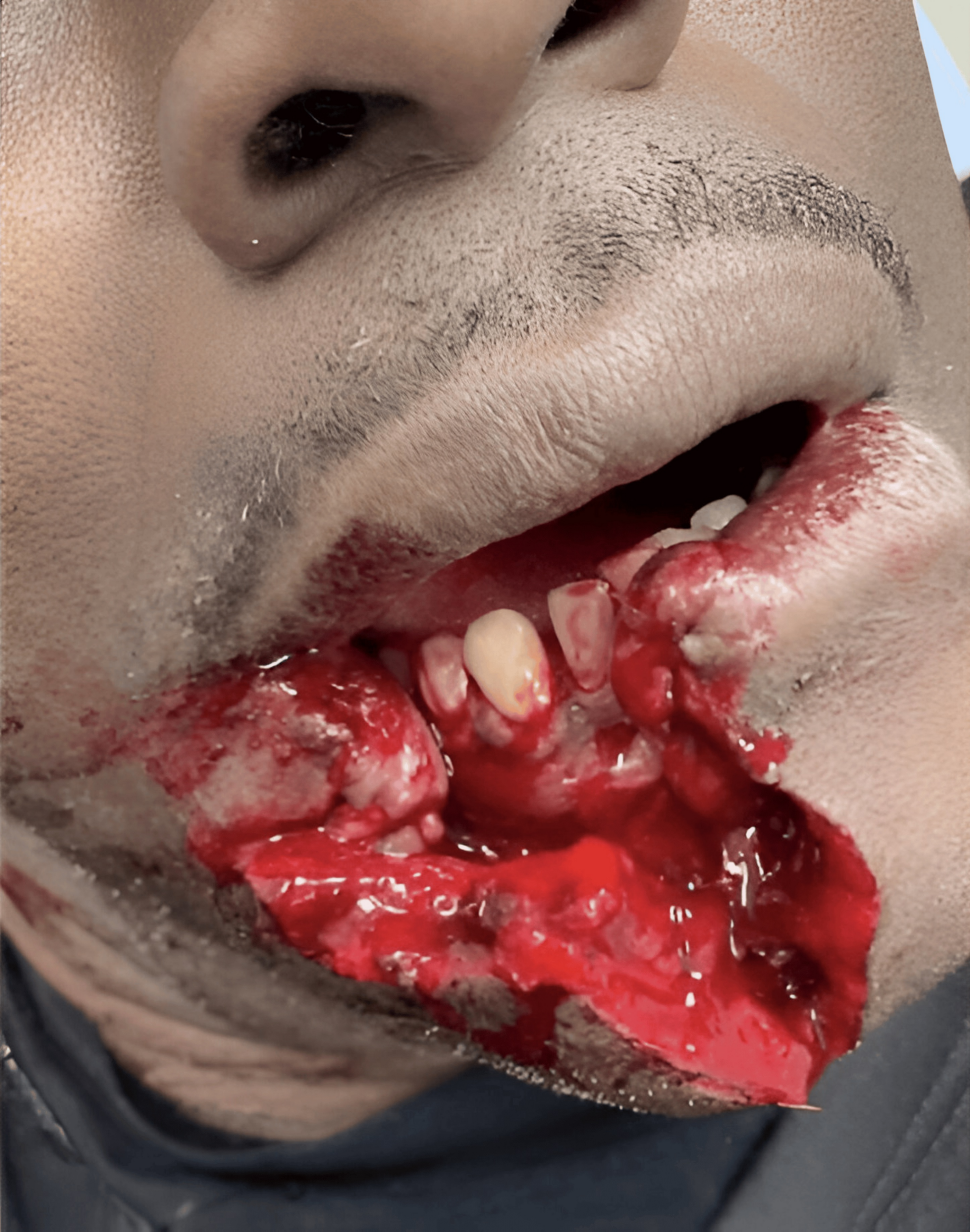
Presentation of dog bite at the emergency department

A 60-year-old male smoker with a history of anemia presented to the emergency department with a complex laceration with loss of tissue to the lower right face due to a dog bite as seen in Figure 1. The patient had been bitten after he had provoked a friend’s dog, resulting in significant bleeding and the loss of part of his lower lip. The avulsion wound was wedge-shaped and accompanied by an approximately 8 cm laceration extending down to the chin and beyond with the mandible bone exposed on presentation. The laceration involved the red vermillion, white border, and loss of a 3 cm x 2 cm area of the lower right lip with shredded edges. The missing area of tissue presented in a plastic bag was deemed viable for a future composite graft after being cleaned and debrided in the emergency department and kept on ice. The tissue sample provided was not traumatically damaged and the edges of the graft were cleaned and debrided such that the tissue was clean enough to use.

The owner of the dog who brought the patient to the hospital reported that the dog was up to date with all vaccinations. Thus, post-exposure rabies prophylaxis was not given to the patient and the animal was quarantined for observation. The patient denied any fever with chills, chest pain, nausea, or vomiting. He also reported being up to date on all vaccinations including tetanus. The emergency department also gave the patient broad-spectrum antibiotics to cover for any bacterial infection before any grafting began. There was no damage to the tongue, teeth, or mandible. Jaw function was preserved and CT was negative for fracture or tooth involvement. Thus, following local anesthesia, the excisional debridement of tissue, skin, subcutaneous tissue, orbicularis oris muscle, and mucosa on the patient’s face was performed after discussing the risks and benefits of this procedure via the emergency medicine department.

Evaluation of the patient by the surgeon for primary wound closure without a skin graft deemed that the defect was too large and a primary wound closure would leave the patient with an unfavorable functional and cosmetic outcome. With primary wound closure, there is a risk of a tight lower lip that fails to create a seal with closure of the mouth, causing the patient to have lifelong drooling, possible speech impediment, and limited function. Upon counseling the patient on both the aesthetics and function of the procedure, it was advised that a composite myomucocutaneous graft of the original tissue presented at the emergency department by the patient would be best at this time and to do a scar revision of the site later if needed. This would prevent any unnecessary cosmetic defects to the patient by grafting skin from another site, using a flap, or reducing the function of the patient’s lower lip by trying to bring the margins of the laceration together at this time.

**Figure 2:**
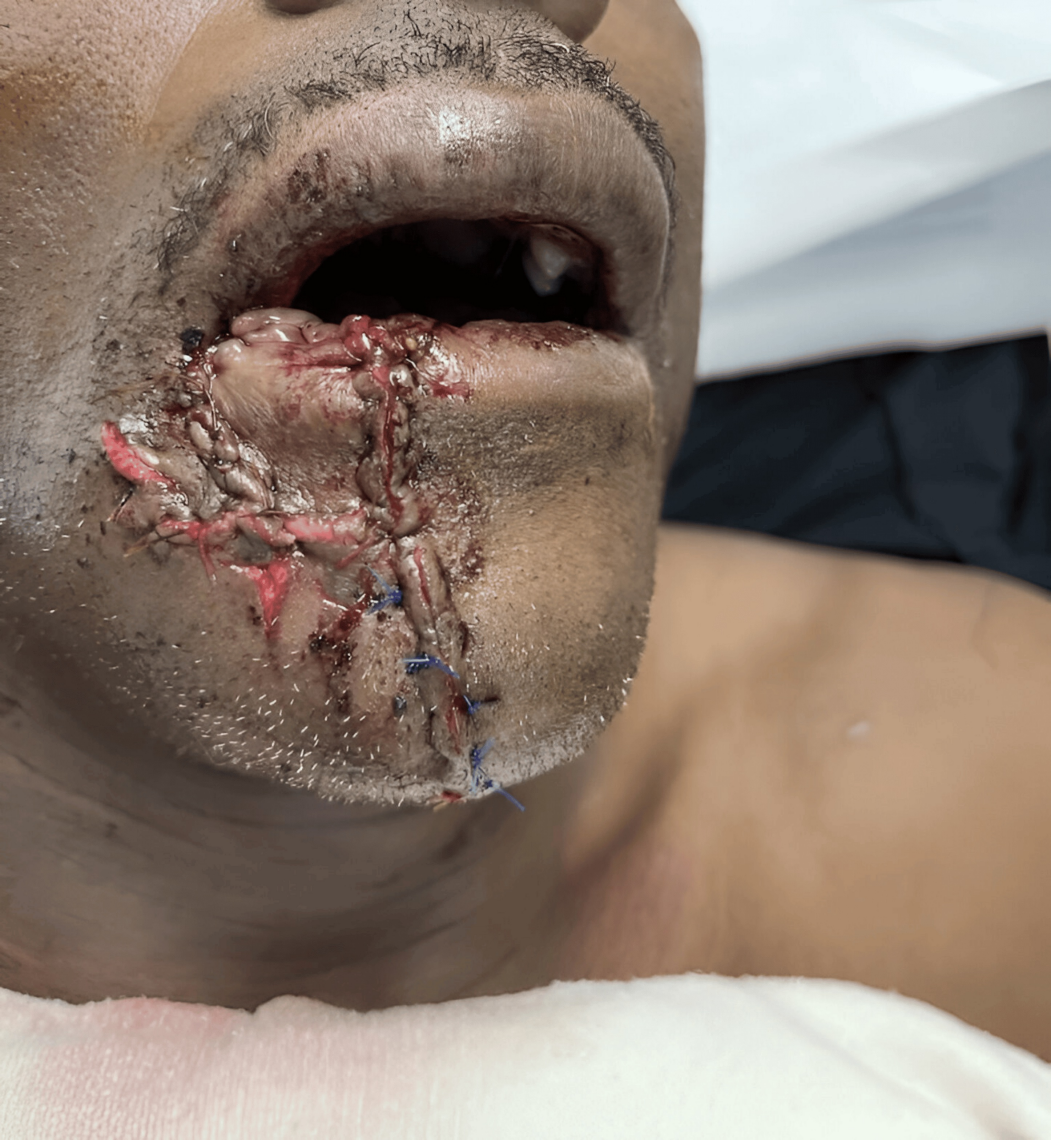
Composite myomucocutaneous graft in the emergency department

The patient agreed to have the tissue grafted on the lower lip, and he was advised that in six to seven months after scar tissue had formed scar revision would be done to repair the graft and approximate the edges of the vermillion border for a more aesthetic appearance. In the emergency department, the preserved skin was reattached via a composite graft as seen in Figure 2. The surgeon was able to mobilize the muscle and mucosa by undermining but could not extensively do so as the angle of the mouth was becoming distorted. The surgeon took into account the patient’s skin color, being aware that individuals with high melanin content can have a higher chance of aesthetic complications such as keloids and hypo or hyperpigmentation of the skin. Thus, it was of utmost importance to limit the amount of incisions and grafts in this patient. The patient was advised to follow up weekly until scar revision could be done.

**Figure 3:**
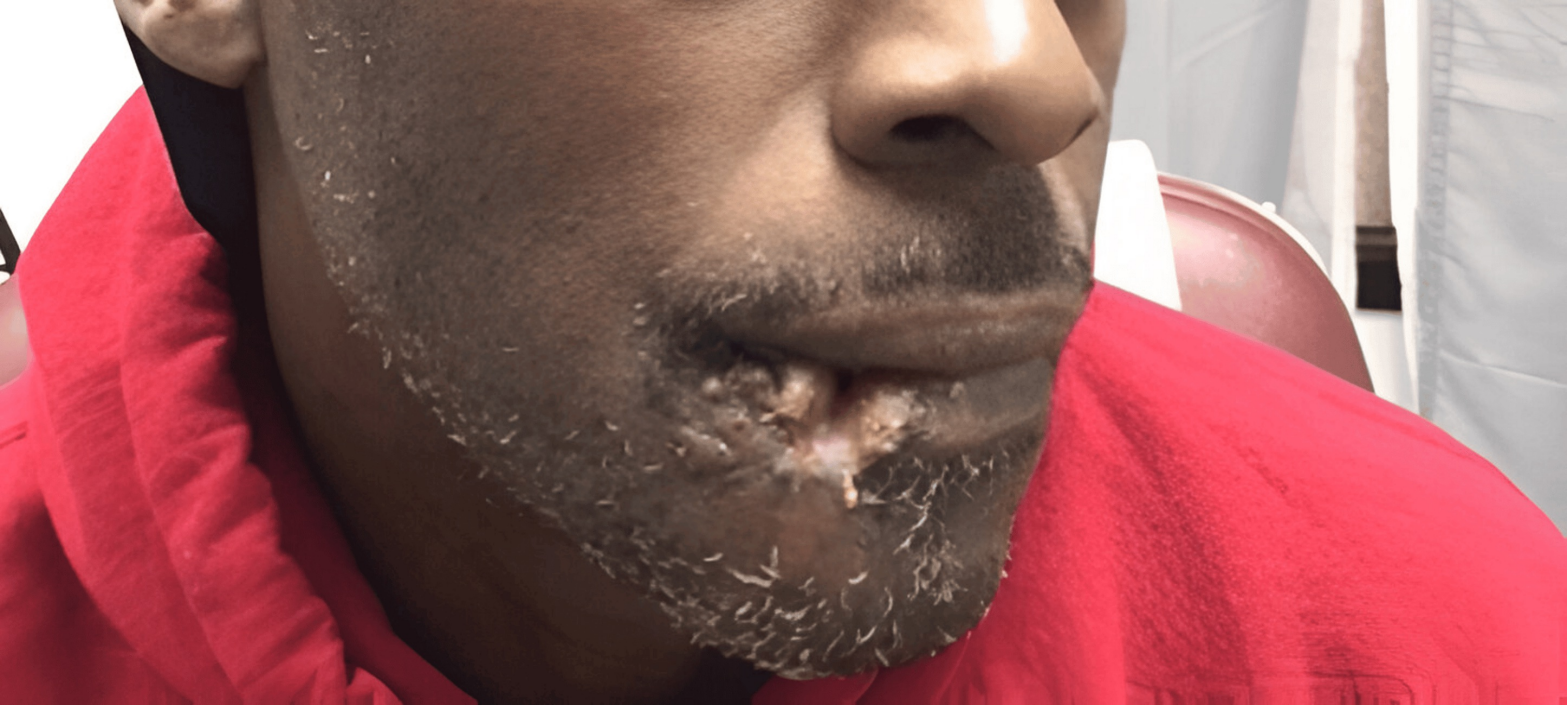
Presentation at the one-month follow-up

At the first outpatient follow-up one week after the injury, the patient presented a repaired laceration with a defect in the lower lip where food and saliva were seen drooling out. The defect measured approximately 3 cm x 2 cm, due to partial failure of the skin graft at the corner of the right lower lip. The surgeon advised to follow up weekly to look into the lesion, allow the scar to contract with scar tissue, and then soften before revision. The healing process one month after the graft can be seen in Figure 3. The nodular defect seen in Figure 3 was attributed to the fact that the tissue originally presented to the emergency department and used for the graft had been retrieved from the dog’s mouth, and the department and surgeon had been unaware of this until after grafting was complete due to the incapacitated state of the patient upon presentation to the emergency department from high-dose hydromorphone for pain management. This slight failure in revascularization of the graft was unanticipated and required further management involving scar revision.

**Figure 4:**
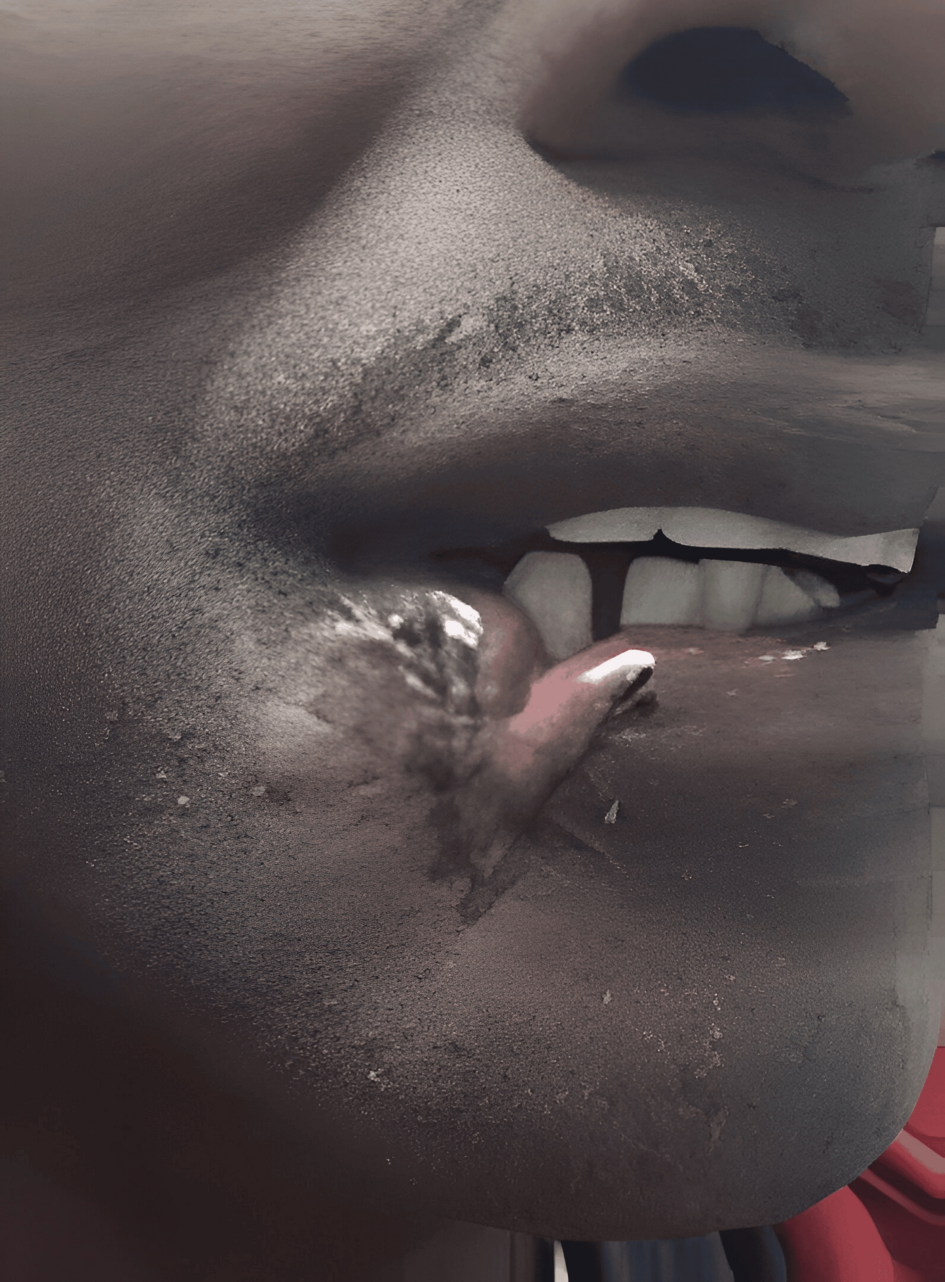
Scar before the revision surgery with pen marking

After six months, the patient had formed enough scar tissue to begin scar revision as ambulatory surgery. The patient presented with pale discolored scars that had become mildly nodular at the junctions of the graft as seen in Figure 4. The patient was marked in a standing position and all incisions were discussed; the risks and benefits were acknowledged by the patient, and he was taken to the operating room where the lower lip was painted and draped. Local anesthesia was infiltrated in and around the scar. The scar tissue was excised with a v-shaped defect of 2.1 cm x 1.5 cm and advancement of flaps in the mucosa and muscle were created via undermining on the medial side of the defect to reduce distortion to the corner of the mouth and oppose the vermilion border properly. Mucosa was sutured with 4-0 Vicryl and 3-0 Vicryl for muscle and 4-0 Prolene for skin. After revision, a sterile dressing was placed. The patient was advised that with this new scar revision, there would be some limited tightness of the lower lip for approximately six months. 

**Figure 5:**
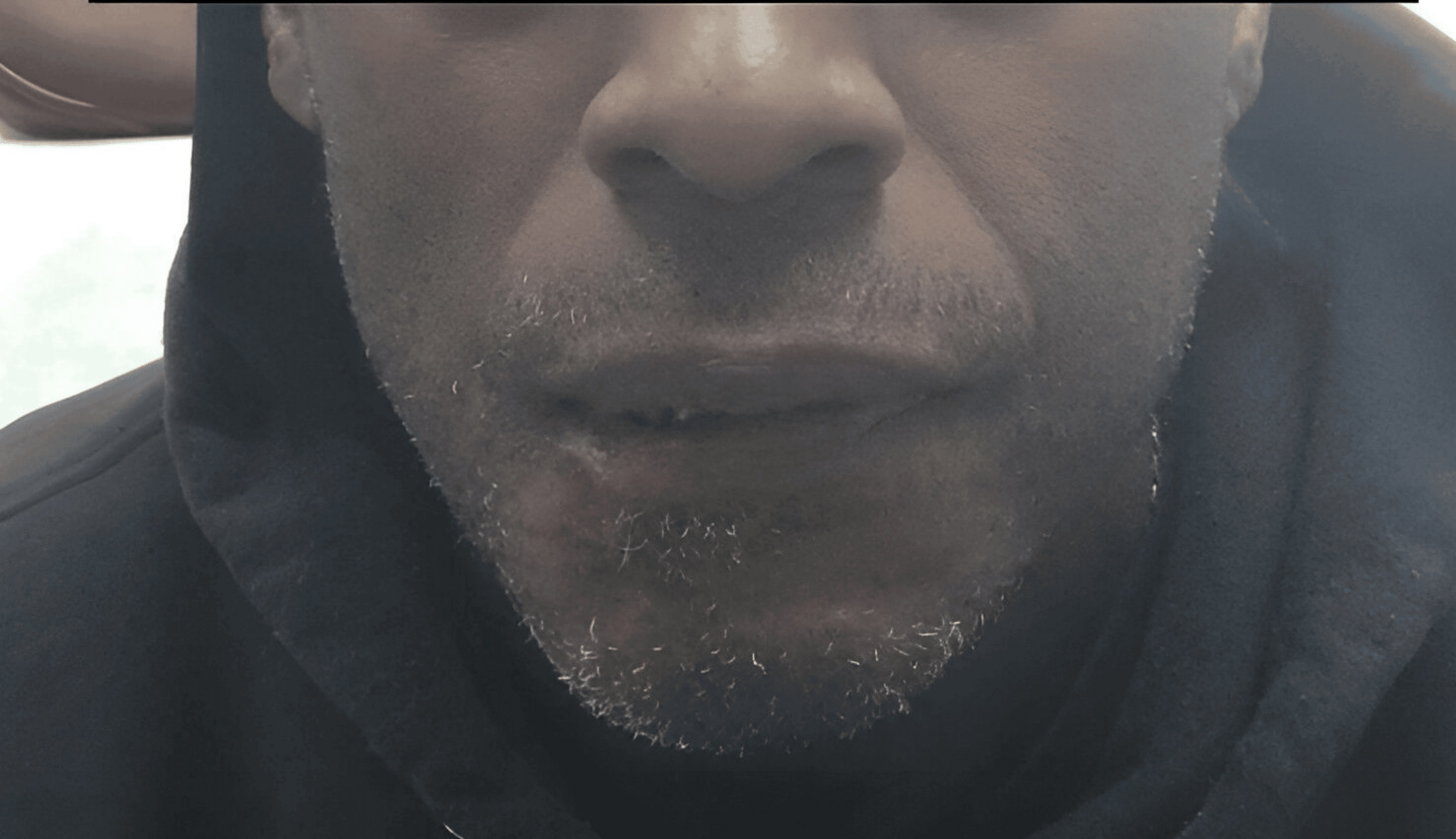
The bite site six months post scar revision

At subsequent follow-ups, the patient was advised to place Neosporin on the scar and he stated that he was well. After suture removal, there were two areas of stitch abscesses, which were deroofed and removed in the office. At the last follow-up almost a year after the original dog bite, the patient was very pleased with the results and was advised to keep massaging the area to manage any swelling. Figure 5 shows the photo of the patient six months post-scar revision.

## Discussion

Dog bites are by far the most commonly reported animal bites in the United States. Medical management protocol for bite wounds includes sanitation and debridement. Irrigation, closure, bacterial culture, appearance of the wound, and timing of medical evaluation after the initial injury all influence the patient outcome after a traumatic animal bite [1]. In one small case series, 57.9% of patients required skin or composite grafting, suturing, incision and drainage, and wound debridement due to oral maxillofacial injuries from dog bites.

Dog bites to the face can be significantly disfiguring and debilitating injuries, causing physical and emotional trauma, and may require long-term therapy for many victims, consisting of multiple surgical revisions and counseling [3]. Primary surgical repair is the most commonly performed procedure for most clinically uninfected facial bite wounds, whereas secondary closure is reserved for certain high-risk or infected wounds. The general practice advises that if more than 1/3 of the lip is damaged, a graft is necessary to close the wound and preserve the function of the patient's mouth, as seen in this case. Avulsive injuries with tissue loss are some of the most difficult cases for definitive management and are also those most likely to require hospitalization [4]. Hence, avulsive injuries to the face require special consideration when it comes to function and aesthetic outcomes.

In accordance with the reconstructive ladder, primary wound closure of facial trauma should be considered first. In cases where primary wound closure is not possible, full-thickness skin grafting is indicated especially on the face; however, the donor site is closed primarily, increasing the chances of multiple scarred areas on the patient. In cases where skin grafting is not possible, flaps are considered [11]. In special circumstances, the use of salvaged tissue in dog bites has been performed. One case study details a dog bite to the central part of the face in which the sample of avulsed tissue contained a combination of skin, muscle, and mucosa. This study detailed how the use of salvaged tissue as a graft caused partial skin necrosis, but revealed healthy vascular tissue immediately below the graft that was used for further reconstruction. This study demonstrated the potential use of salvaged tissue as a bridge to build healthy tissue underneath that can be used for future reconstruction with a better cosmetic and functional result [12]. This concept that healthy tissue would emerge from the graft healing process was employed in this case to avoid unnecessary donor grafting or management via facial flap. Additionally, the availability and quality of the salvaged tissue was assessed and this was taken into consideration as well. 

Among available treatments for avulsion dog injuries, grafting and reconstruction are the most common for dog bites to the face. For cosmetic reasons, revision surgery is indicated for hypertrophic scarring seen in patients post-injury, which is most common in patients with high melanin content [3]. While many skin reduction techniques have been described, these can be complicated by poor scars such as hypertrophic or keloid scars in patients with high melanin content in their skin [13]. In this case, while the graft was initially successful, partial failure was observed during the first follow-up, particularly at the corner of the right lower lip. This was attributed to a slight issue with graft revascularization, which was most likely compounded by the use of tissue recovered from the dog’s mouth, a source of potential contamination. Although the graft itself was not traumatically damaged, the fact that it was retrieved from an animal’s oral cavity introduced additional risks, particularly in terms of vascular compromise. The nodular defect that developed at one month was due to this partial graft failure, necessitating further management and anticipated scar revision.

An important aspect of this case was the consideration of the patient’s skin type. As someone with dark skin, our patient was at increased risk for hypertrophic scarring and keloid formation, both of which are common complications following facial reconstructive surgery in individuals with high melanin content. The surgeon took extra care to limit the number of incisions and avoid extensive flap dissection, which could exacerbate the risk of poor scar formation. The decision to use the graft tissue retrieved from the dog, while not without risk, was made in part to avoid further scarring from donor sites, such as from the upper lip or other areas that might have complicated the aesthetic results. Although hypertrophic scarring and keloid formation are known complications in patients with melanated skin, the use of meticulous surgical techniques and careful scar management with the use of Neosporin and massaging the scar contributed to an excellent final result.

## Conclusions

This report highlights the importance of individualized treatment planning for complex facial trauma, particularly in patients with high melanin content. The choice of using a composite graft from the original tissue was a critical factor in preserving both function and aesthetics. While there were some challenges with graft revascularization, the long-term outcome was favorable. This demonstrates that with careful surgical planning, the risks of further disfigurement or functional loss can be minimized. The report also emphasizes the need for greater awareness and research on the management of facial injuries in individuals with melanated skin, as they present unique challenges in terms of scar formation and aesthetic outcomes. Thus, the careful consideration of both functional and aesthetic concerns, as well as the recognition of skin type and its potential complications, were key to achieving a positive result in this patient. The report underscores the need for tailored approaches to surgical management, particularly in high-risk patients, to ensure optimal outcomes both functionally and cosmetically.
